# Lipid-Modulating Effects of *Sargassum fulvellum* Fermented by *Lactococcus lactis* KCCM12759P and *Leuconostoc mesenteroides* KCCM12756P in Ovariectomized Mice

**DOI:** 10.3390/nu17152527

**Published:** 2025-07-31

**Authors:** Hyun-Sol Jo, Young-Eun Cho, Sun-Mee Hong

**Affiliations:** 1 Marine Industry Research Institute for East Sea Rim, 22 Haeyanggwahak-gil, Uljin 36315, Gyeongsangbuk-do, Republic of Korea; 2 Korea Food Research Institute, Iseo-myeon, Wanju 55365, Jeollabuk-do, Republic of Korea; 3 Department of Food and Nutrition, Gyeongkuk National University, Andong 36729, Gyeongsangbuk-do, Republic of Korea

**Keywords:** *Sargassum*, fermentation, estrogen, dyslipidemias, vitamin K2

## Abstract

**Background/Objectives**: Estrogen deficiency contributes to dyslipidemia and visceral adiposity, increasing cardiovascular risk in postmenopausal women. *Sargassum fulvellum* (Sf), a brown seaweed rich in bioactive compounds, possesses lipid-regulating properties that may be enhanced by lactic acid bacteria fermentation. This study aimed to evaluate the effects of fermented *S. fulvellum* (SfLlLm), prepared using *Lactococcus lactis* and *Leuconostoc mesenteroides*, on lipid metabolism and adipose tissue remodeling in an ovariectomized (OVX) mouse model of estrogen deficiency. **Methods**: Female C57BL/6 mice underwent ovariectomy and were fed an AIN-76A diet supplemented with either unfermented Sf or SfLlLm for eight weeks. Sham-operated and 17β-estradiol-treated OVX groups served as controls. Serum lipid levels—total cholesterol, triglycerides, LDL-C, and HDL-C—were assessed, and histological analysis of visceral adipose tissue was conducted to evaluate adipocyte morphology. **Results**: OVX-induced estrogen deficiency led to increased total cholesterol, triglycerides, and LDL-C, along with hypertrophic changes in visceral adipocytes. Supplementation with fermented *Sargassum fulvellum* (SfLlLm) markedly improved these parameters, reducing total cholesterol by 6.7%, triglycerides by 9.3%, and LDL-C by 52.9%, while increasing HDL-C by 17.5% compared to the OVX controls. SfLlLm also normalized visceral adipocyte size and distribution. These effects were comparable to or exceeded those of 17β-estradiol treatment. **Conclusions**: Fermented SfLlLm ameliorated dyslipidemia and visceral adiposity under estrogen-deficient conditions. These findings support its potential as a functional dietary intervention for managing postmenopausal lipid disorders and associated metabolic complications.

## 1. Introduction

Menopause is accompanied by a decline in circulating estrogen levels, which contributes to a range of metabolic disturbances, including dyslipidemia, hepatic steatosis, and visceral adiposity. These changes increase the risk of developing cardiovascular diseases, non-alcoholic fatty liver disease, and obesity-related complications [1,2]. The ovariectomized (OVX) mouse model, which mimics the hormonal milieu of postmenopausal women, is widely employed in preclinical research to evaluate the efficacy of dietary and pharmacological interventions targeting estrogen deficiency-associated metabolic dysfunction [3].

Marine algae have recently garnered substantial interest as sustainable and potent sources of functional food ingredients [4,5,6]. As photosynthetic organisms, seaweeds possess an impressive repertoire of bioactive compounds, including polysaccharides (e.g., fucoidan, laminarin), phlorotannins, carotenoids (e.g., fucoxanthin), soluble fibers, and essential micronutrients such as vitamins A, C, E, and K [4,5,7]. These components contribute to diverse biological activities, including antioxidant, anti-inflammatory, lipid-lowering, anti-obesity, and hepatoprotective effects. Consequently, seaweeds are being increasingly explored for their applications in the food, nutraceutical, and pharmaceutical industries. Additionally, the marine ecosystem harbors a vast diversity of underutilized algae species with unique metabolic profiles and therapeutic potential [4,5,7]. Among these, *Sargassum fulvellum*, an edible brown macroalga traditionally consumed in East Asia, is recognized for its health-promoting properties [5,8]. Rich in phylloquinone (vitamin K1), polyphenols, and other antioxidative constituents, *S. fulvellum* reportedly exerts antioxidant, anti-inflammatory, and osteoprotective effects [5,8,9,10]. However, its functional potential in metabolic health remains underexplored due to the low bioavailability of its active compounds and the limited understanding of their mechanisms of action.

While *Sargassum fulvellum* has been primarily studied for its benefits in bone health—attributed to its anti-inflammatory and antioxidant actions—its relevance to metabolic regulation, particularly lipid metabolism under estrogen-deficient conditions, remains largely unexplored. Menopause-associated dyslipidemia and adipose tissue dysfunction share underlying pathophysiological mechanisms with osteoporosis, including chronic low-grade inflammation, oxidative stress, and impaired mitochondrial function. These overlapping pathways suggest that interventions promoting bone health may exert systemic metabolic benefits as well.

Among the promising candidates for such intervention are menaquinones (vitamin K2 homologs), which are produced during lactic acid bacterial fermentation. These compounds have been shown to support bone mineralization and to play emerging roles in lipid metabolism and vascular homeostasis by suppressing inflammatory cytokines, enhancing mitochondrial efficiency, and modulating the expression of genes involved in lipid transport and oxidation [11,12].

Fermentation has emerged as a promising strategy for enhancing the nutritional and functional value of plant-based ingredients. Lactic acid bacteria (LAB), particularly *Lactococcus lactis* (*L. lactis*) and *Leuconostoc mesenteroides* (*L. mesenteroides*), have demonstrated the ability to not only improve the digestibility and palatability of plant matrices but also biosynthesize menaquinones (vitamin K2), a class of fat-soluble vitamins known to support bone, liver, and lipid metabolism [11,13,14]. Notably, vitamin K2 (menaquinone-4, -7, and -9) has been shown to modulate mitochondrial activity and inflammatory signaling, suggesting its potential ability to ameliorate estrogen deficiency-induced metabolic disturbances [12,15].

Building on this mechanistic rationale, previous work has shown that *S. fulvellum* fermented with *L. lactis* KCCM12759P and *L. mesenteroides* KCCM12756P significantly enhances the production of long-chain menaquinones and increases antioxidant and anti-inflammatory capacity [10]. Given the overlapping mechanisms among inflammation, mitochondrial dysfunction, and lipid dysregulation observed in menopause, the fermented form (SfLlLm) may hold promise as a multifunctional dietary intervention. While prior research has highlighted its bone-protective properties in estrogen-deficient animal models [10], its potential role in regulating lipid metabolism, preventing hepatic lipid accumulation, and remodeling adipose tissue remains to be elucidated [16,17]. In addition to metabolic dysfunction, estrogen deficiency has been associated with adverse psychosocial outcomes and reduced quality of life. Polycystic ovary syndrome (PCOS), a condition characterized by hormonal and metabolic dysregulation, shares features similar to those of postmenopausal states. A study by Panico et al. [18] emphasized the physical and psychological burden of such conditions, highlighting the need for functional interventions that address both metabolic health and overall well-being.

Therefore, the aim of this study was to investigate the effects of *S. fulvellum* and its LAB-fermented derivative (SfLlLm) on lipid profiles, hepatic steatosis, and visceral adipose tissue remodeling in ovariectomized (OVX) mice as a model of estrogen-deficient metabolic dysfunction. This study also contributes to the broader understanding of fermented marine bioresources as candidate ingredients for the dietary management of postmenopausal health.

## 2. Materials and Methods

### 2.1. Preparation of fermented S. fulvellum

**Figure 1:**
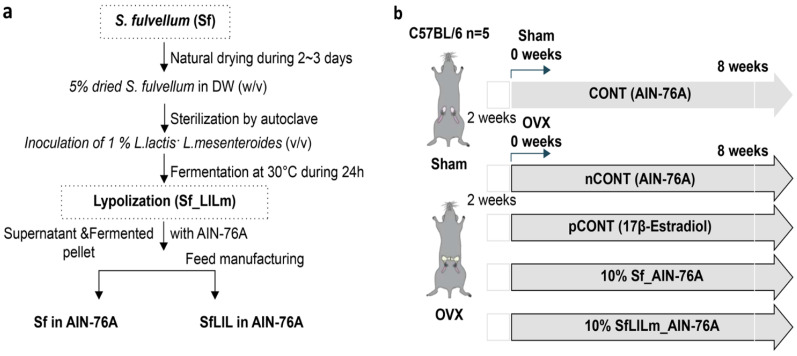
Sample preparation and experimental timeline for this in vivo study using *Sargassum fulvellum* (Sf). (**a**) Flowchart illustrating the feed preparation process using Sf and its fermented form (Sf_LlLm); (**b**) Schematic overview of the animal experiment involving sham-operated (CONT) and ovariectomized (OVX) C57BL/6 mice. Mice were assigned to the following groups (*n* = 5 per group): CONT, sham-operated mice fed a standard AIN-76A diet; nCONT, OVX mice fed the AIN-76A diet; pCONT, OVX mice fed AIN-76A supplemented with 17β-estradiol; Sf, OVX mice fed AIN-76A containing 10% Sf; and SfLlLm, OVX mice fed AIN-76A containing 10% SfLlLm. All treatments were administered for 8 weeks following a 2-week recovery period after sham or OVX surgery.

Dried *S. fulvellum* was obtained from a domestic commercial supplier (Parajeju, Jeju, Republic of Korea; http://www.parajeju.com) and ground finely into powder. As illustrated in Figure 1a, fermentation was performed using *L. lactis* KCCM12759P and *L. mesenteroides* KCCM12756P, following the protocol described in a previous study [10]. Each strain was inoculated at 1% (*v/v*) into a sterile suspension of *S. fulvellum* (5% *w/v* in distilled water), which had been autoclaved to ensure sterility. The mixture was incubated at 30 °C for 24 h under static conditions. After fermentation, the culture was centrifuged to separate solids and liquids, which were then recombined and freeze-dried to produce the fermented product (SfLlLm). For the non-fermented control (Sf), dried *S. fulvellum* powder was directly incorporated into the experimental diet without undergoing fermentation.

### 2.2. Ethics Statement

All animal procedures, including the OVX-induced osteoporosis mouse model and subsequent experimental protocols, were reviewed and approved by the Institutional Animal Care and Use Committee of Andong National University (Approval No. 2024-4-1111-06-01; Approval Date: 11 November 2024).

### 2.3. Experimental Animals and Design

As shown in Figure 1b, female C57BL/6 mice (8 weeks old) were purchased from Orient Bio Inc. (Seongnam, Republic of Korea) and acclimated for 1 week under standard laboratory conditions (22 ± 2 °C, 12 h light/dark cycle, 55 ± 10% relative humidity). Anesthesia was induced using tribromoethanol, after which the mice were subjected either to sham surgery (without ovariectomy) or to bilateral OVX to establish a model of estrogen deficiency-induced menopause. Precisely 2 weeks post-surgery, the mice were randomly assigned to five experimental groups (*n* = 5 per group): CONT (sham-operated, AIN-76A diet); nCONT (OVX, AIN-76A); pCONT (OVX, AIN-76A + 17β-estradiol at 0.01%); Sf (OVX, AIN-76A + 10% *S. fulvellum*); and SfLlLm (OVX, AIN-76A + 10% fermented *S. fulvellum*). Throughout the 8-week dietary intervention, body weight was recorded weekly to monitor metabolic changes. At the end of the study, the mice were fasted overnight and then euthanized under anesthesia. All surgical procedures were performed under anesthesia using tribromoethanol to minimize pain during the ovariectomy. Postoperative analgesia was not provided, as minimal discomfort was expected under tribromoethanol anesthesia, and no signs of pain or distress were observed. However, we acknowledge that analgesia is recommended and will apply this in future studies. However, no specific post-operative analgesics or pain monitoring protocols were described. No adverse events were observed, and all animals completed the study. Humane endpoints were not established, and no signs of distress or morbidity were reported during the 8-week dietary intervention. Blood and tissue samples were collected for biochemical and histological analyses. No inclusion or exclusion criteria were explicitly established a priori, and no animals or data points were excluded from the analysis in any experimental group. All animals assigned to the five groups (*n* = 5 per group) completed the study and were included in the final data analysis. Animals were randomly assigned to experimental groups following a 2-week recovery from sham or ovariectomy surgery. However, the method of random sequence generation was not specified. No specific strategies were described to control for potential confounding variables such as cage location, handling order, or measurement sequence. Randomization was performed using a simple random number generator in Microsoft Excel to assign animals to each treatment group. However, blinding was not implemented during sample collection or analysis owing to personnel limitations, which may introduce detection bias. This placement ensures it is reported transparently alongside other design-related details (e.g., randomization, group allocation, ethical approval). Outcome assessments, including biochemical analyses and histological evaluations, were not performed under blinded conditions. All analyses were conducted by researchers aware of the group allocations.

### 2.4. Tissue and Serum Collection

Liver, epididymal fat, and retroperitoneal fat pads were carefully excised, rinsed with sterile saline (0.75% NaCl; Sigma-Aldrich, St. Louis, MO, USA), blotted dry using filter paper (No. 2, Advantec, Tokyo, Japan), and weighed using an analytical balance (GR-202, A&D Company, Tokyo, Japan). Blood was collected via cardiac puncture under anesthesia, and serum was separated by centrifugation at 3000× *g* for 10 min at 4 °C using a refrigerated centrifuge (Gyrozen 1236R, Daejeon, Republic of Korea). The serum was aliquoted into microtubes (Eppendorf, Hamburg, Germany) and stored at −80 °C until further analysis.

### 2.5. Serum Biochemical Analysis

Serum concentrations of triglycerides (TG; Triglyceride Quantification Kit, BioVision, Seoul, Republic of Korea), total cholesterol (TC; Total Cholesterol Colorimetric Assay Kit, Abcam, Cambridge, UK), high-density lipoprotein cholesterol (HDL-C; Abcam, Cambridge, UK), low-density lipoprotein cholesterol (LDL-C; Abcam, Cambridge, UK), aspartate aminotransferase (AST; Abcam, Cambridge, UK), and alanine aminotransferase (ALT; Abcam, Cambridge, UK) were determined using commercial enzymatic colorimetric assay kits, according to the manufacturers’ instructions. Absorbance was measured using a microplate reader (SpectraMax iD3, Molecular Devices, San Jose, CA, USA).

### 2.6. Histological Analysis

Liver and epididymal adipose tissues were fixed in 10% neutral-buffered formalin (Sigma-Aldrich, St. Louis, MO, USA) for 24 h. The fixed tissues were then dehydrated through a graded ethanol series (70%, 80%, 90%, 95%, and 100%; 10 min each; Sigma-Aldrich), cleared in xylene (Sigma-Aldrich), and embedded in paraffin. Paraffin blocks were sectioned at a thickness of 5 μm using a rotary microtome (RM2235, Leica Biosystems, Wetzlar, Germany). The sections were stained with hematoxylin and eosin (H&E; Merck, Darmstadt, Germany) according to standard histological protocols. Histological features, including hepatic lipid accumulation and adipocyte morphology, were examined using a light microscope (BX51, Olympus, Tokyo, Japan). Representative images were captured at 200× magnification using a digital camera (DP74, Olympus, Tokyo, Japan). The primary outcome was not explicitly defined, and no formal sample size calculation was conducted. A sample size of *n* = 5 per group was used based on precedents from similar preclinical studies.

### 2.7. Statistical Analyses

All data are presented as mean ± standard deviation (SD). Statistical analyses were performed using SPSS Statistics for Windows, version 27.0 (IBM Corp., Armonk, NY, USA; https://www.ibm.com/products/spss-statistics, accessed on 30 June 2024). Group differences were assessed using one-way analysis of variance, followed by Tukey’s post hoc test. A *p*-value < 0.05 was considered statistically significant. The sample size (*n* = 5 per group) was based on similar exploratory studies using OVX mice. Although no formal a priori power calculation was performed, post hoc analysis indicated sufficient power (>80%) for key metabolic outcomes. We acknowledge the small sample size as a limitation and recommend larger, adequately powered studies in the future. No formal study protocol was pre-registered.

## 3. Results and Discussion

### 3.1. Effects of Fermented S. fulvellum on Body and Organ Weights in OVX Mice

**Figure 2:**
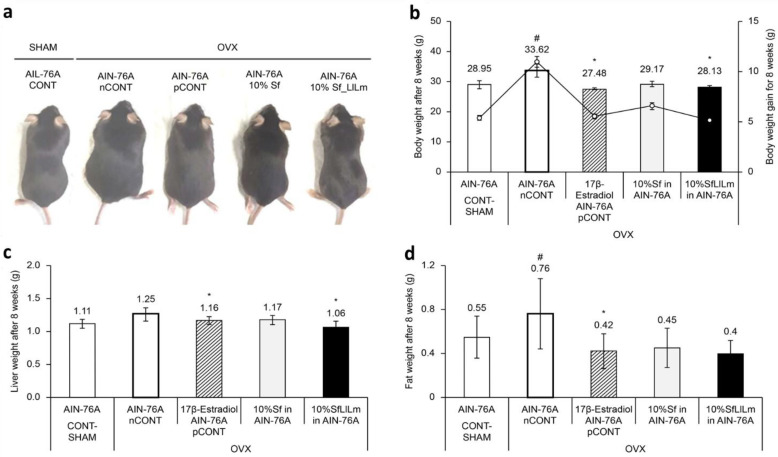
Effects of *Sargassum fulvellum* (Sf) and its fermented form (SfLlLm) on body, liver, and fat weights in ovariectomized (OVX) mice. (**a**) Representative dorsal images of mice from each group after 8 weeks of dietary intervention. (**b**) Final body weight (bar graph) and weight gain (line graph) in mice. (**c**) Relative liver weight normalized to body weight. (**d**) Relative fat weight, including epididymal and retroperitoneal fat, normalized to body weight. Data are presented as mean ± standard error of the mean (*n* = 5). # *p* < 0.05 vs. CONT (sham-operated control); * *p* < 0.05 vs. nCONT (OVX control), indicating significant effects of 17β-estradiol, Sf, or SfLlLm treatment.

Representative dorsal images of mice from each group after 8 weeks of dietary intervention are shown in Figure 2a. Mice in the OVX control group (nCONT) exhibited markedly increased body size and visible abdominal expansion compared to the sham-operated group (CONT), reflecting the typical weight gain and fat accumulation associated with estrogen deficiency. In contrast, the Sf and SfLlLm groups displayed visibly smaller abdominal girth and overall body size relative to nCONT, suggesting an amelioration of OVX-induced metabolic alterations. Notably, the SfLlLm group exhibited body contours closely resembling those of the pCONT group (OVX + 17β-estradiol), indicating potential protective effects of the fermented product against adiposity and weight gain in OVX mice.

These visual findings were consistent with the quantitative measurements. As shown in Figure 2b, administration of 10% Sf or SfLlLm for 8 weeks significantly suppressed body weight gain compared to the nCONT. Additionally, both Sf and SfLlLm led to notable reductions in liver weight and retroperitoneal fat pad weight (Figure 2c,d). Among the dietary groups, SfLlLm showed the most substantial improvements in these metabolic indicators. Although the SfLlLm group exhibited slightly greater reductions in cumulative body weight, liver weight, and fat mass than pCONT, these findings should be interpreted cautiously given the exploratory nature of the study and limited sample size. These results align with previous reports demonstrating the efficacy of dietary seaweed in mitigating obesity-related outcomes in estrogen-deficient animal models [17,19].

The enhanced metabolic efficacy of SfLlLm compared to unfermented *S. fulvellum* may be attributed to the formation of fermentation-enriched bioactives, including long-chain menaquinones (e.g., MK-4, MK-7, MK-9), short-chain fatty acids (SCFAs), and microbial exopolysaccharides produced by *L. lactis* and *L. mesenteroides* [10,20,21,22]. These metabolites are reported to influence antioxidant activity, lipid metabolism, mitochondrial function, and systemic inflammation, which are central to adiposity and metabolic regulation [23]. However, while these hypotheses are supported by prior research, molecular validation (e.g., gene or protein expression analyses) was not conducted in the present study. Therefore, the involvement of these compounds in mediating the observed outcomes remains speculative and warrants further investigation.

While hormone replacement therapy (HRT) restores systemic estrogen levels to ameliorate menopausal symptoms, the metabolic benefits of SfLlLm appear to be exerted through non-hormonal pathways involving fermentation-derived bioactives. This distinction may offer potential advantages in safety and tolerability, particularly for populations for whom HRT is contraindicated. Nonetheless, the long-term efficacy and safety of SfLlLm require validation in future preclinical and clinical studies.

Given the growing demand for safe, food-based interventions to address menopausal metabolic dysfunction, fermented *S. fulvellum* represents a promising candidate as a functional dietary supplement to manage weight gain and hepatic steatosis in postmenopausal populations.

### 3.2. Hepatoprotective and Hypolipidemic Effects of Fermented S. fulvellum

**Figure 3:**
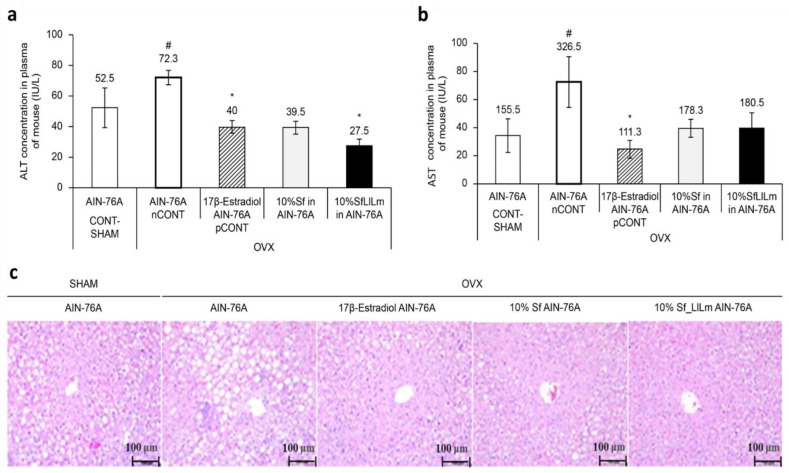
Effects of *Sargassum fulvellum* (Sf) and its fermented form (SfLlLm) on hepatic lipid accumulation and serum biochemical parameters in ovariectomized (OVX) mice. (**a**) Serum alanine aminotransferase (ALT) levels. (**b**) Serum aspartate aminotransferase (AST) levels. (**c**) Representative histological images of liver tissues stained with hematoxylin and eosin (H&E), showing lipid accumulation in each group (scale bar = 100 µm). Data are expressed as mean ± standard error of the mean (*n* = 5). # *p* < 0.05 vs. CONT (sham-operated control); * *p* < 0.05 vs. nCONT (OVX control), indicating significant effects of 17β-estradiol, Sf, or SfLlLm treatment.

As shown in Figure 3a, ALT levels were significantly elevated in the OVX control group (nCONT) compared to the sham-operated group (CONT), indicating hepatic dysfunction associated with estrogen deficiency. However, administration of either Sf or SfLlLm for 8 weeks markedly reduced ALT levels. Among the treatment groups, SfLlLm exhibited the most pronounced ALT-lowering effect, comparable to that of the 17β-estradiol-treated group (pCONT). Similarly, Figure 3b shows that serum AST levels were also significantly increased in the nCONT group relative to CONT. Both Sf and SfLlLm effectively reduced AST levels, with SfLlLm again demonstrating greater efficacy than the unfermented form. These biochemical improvements were further corroborated by histological analysis. As presented in Figure 3c, liver sections stained with H&E revealed prominent lipid droplet accumulation in the livers of the nCONT mice, indicative of hepatic steatosis. In contrast, mice treated with Sf or SfLlLm exhibited markedly fewer lipid vacuoles, suggesting reduced hepatic fat deposition. Notably, the SfLlLm group displayed a histological profile most similar to that of pCONT, indicating a strong protective effect against OVX-induced fatty liver. Collectively, these findings demonstrate that fermented *S. fulvellum* not only improves serum lipid profiles but also alleviates hepatic lipid accumulation and injury in estrogen-deficient mice. In particular, the normalization of ALT and AST levels suggests hepatoprotective effects, which may be partially mediated by fermentation-enriched bioactive compounds such as vitamin K2.

Vitamin K2 reportedly attenuates hepatic inflammation and fibrosis by modulating oxidative stress, mitochondrial function, and expression of proinflammatory cytokines [11]. Given that lactic acid fermentation of *S. fulvellum* significantly increases MK-4, MK-7, and MK-9 levels [10], these menaquinones may play a key role in restoring liver enzyme balance and mitigating liver damage. Additionally, SCFAs and microbial exopolysaccharides produced during fermentation are also known to improve hepatic lipid metabolism and reduce the steatotic burden [21,22]. Importantly, the hepatoprotective and lipid-lowering effects of SfLlLm were comparable to—or in some cases exceeded—those of 17β-estradiol. This suggests that fermented *S. fulvellum* may exert its metabolic benefits through estrogen-independent mechanisms while offering a favorable safety profile. These findings are particularly relevant, considering the well-documented risks associated with long-term HRT, such as stroke and thromboembolism [24,25]. Therefore, SfLlLm may serve as a promising food-derived alternative for managing dyslipidemia, hepatic steatosis, and liver enzyme imbalance in postmenopausal women.

### 3.3. Effects of Fermented S. fulvellum on Serum Lipid and Adipose Tissue

**Figure 4:**
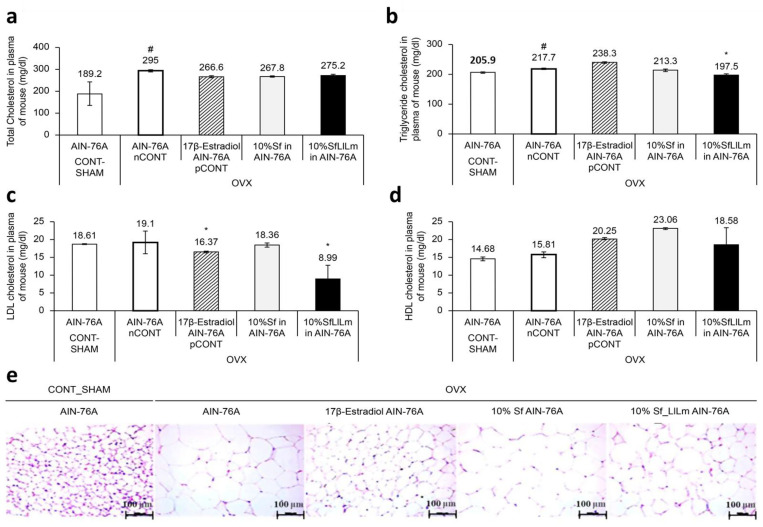
Effects of *Sargassum fulvellum* (Sf) and its fermented form (SfLlLm) on serum lipid levels and adipocyte morphology in ovariectomized (OVX) mice. (**a**) Serum total cholesterol (TC) levels. (**b**) Serum triglyceride (TG) levels. (**c**) Serum low-density lipoprotein cholesterol (LDL-C) levels. (**d**) Serum high-density lipoprotein cholesterol (HDL-C) levels. (**e**) Representative histological images of epididymal adipose tissue stained with hematoxylin and eosin (H&E), showing adipocyte size and distribution (scale bar = 100 µm). Data are presented as mean ± standard error of the mean (*n* = 5). # *p* < 0.05 vs. CONT (sham-operated control); * *p* < 0.05 vs. nCONT (OVX control), indicating significant effects of 17β-estradiol, Sf, or SfLlLm treatment.

As shown in Figure 4a,b, serum TC and TG levels were significantly elevated in the OVX control group (nCONT) compared to the sham-operated group (CONT), reflecting dyslipidemia induced by estrogen deficiency. Dietary supplementation for 8 weeks with either Sf or SfLlLm markedly reduced TC and TG levels. Among the treatments, SfLlLm exhibited lipid-lowering effects, comparable to those of 17β-estradiol (pCONT). Regarding lipoprotein profiles (Figure 4c,d), LDL-C levels were moderately elevated in the nCONT group, indicating impaired lipid transport and increased cardiovascular risk. All treatment groups—Sf, SfLlLm, and 17β-estradiol—significantly reduced LDL-C levels, with SfLlLm showing the most notable reduction. HDL-C levels were also significantly increased following all treatments. Interestingly, unfermented Sf led to the highest HDL-C elevation, suggesting that native bioactive compounds within *S. fulvellum* may promote reverse cholesterol transport. Histological analysis of epididymal adipose tissue (Figure 4e) revealed that the nCONT group displayed enlarged, irregularly distributed adipocytes typical of visceral fat hypertrophy. In contrast, both Sf and SfLlLm treatments led to smaller, more uniform adipocytes. The SfLlLm group, in particular, exhibited an adipocyte morphology closely resembling that of the pCONT group, indicating a reversal of OVX-induced adipose remodeling. These data collectively suggest that SfLlLm exerts beneficial effects on systemic lipid homeostasis and adipose tissue architecture under estrogen-deficient conditions. The simultaneous reduction of LDL-C and normalization of adipocyte morphology, alongside increased HDL-C levels, underscore the dual regulatory potential of SfLlLm on lipid transport and storage.

The enhanced metabolic efficacy of SfLlLm appears to be associated with fermentation-derived metabolites, including vitamin K2 homologs (menaquinones MK-4, MK-7, MK-9), short-chain fatty acids (SCFAs), and microbial exopolysaccharides. These compounds are known to modulate adipogenesis, promote lipid oxidation, and attenuate inflammation in adipose tissue [21,22]. In particular, menaquinones have demonstrated potential for reducing adipocyte hypertrophy, improving insulin sensitivity, and suppressing proinflammatory cytokine expression [10], which aligns with the histological and biochemical improvements observed in this study. While unfermented *S. fulvellum* also improved the lipid profiles, its effects were relatively modest. This may be due to its native content of marine bioactives such as fucoidans, polyphenols, and sterols, which are associated with cholesterol metabolism and vascular health.

Our findings support the hypothesis that fermentation enhances the lipid-modulating efficacy of *S. fulvellum* under estrogen-deficient conditions, likely by generating additional or synergistic bioactive compounds. This aligns with prior reports on the metabolic benefits of vitamin K2, SCFAs, and fermented seaweed components. Nevertheless, several limitations should be acknowledged. First, the small sample size (*n* = 5 per group) may limit the statistical power and generalizability. Second, the absence of blinding during data collection and histological evaluation could introduce observer bias. Third, although the OVX mouse is a well-established model of postmenopausal metabolic dysfunction, it does not fully recapitulate the complexity of human physiology. Finally, mechanistic pathways were not directly evaluated in this study, and future investigations should include molecular analyses to elucidate the underlying mechanisms. Additionally, the outcome assessments were not blinded, which may introduce potential observer bias. Although objective assay kits and standardized histological protocols were used, the lack of blinding remains a methodological limitation.

Despite these limitations, SfLlLm effectively mitigated dyslipidemia and adipose tissue hypertrophy induced by estrogen deficiency in OVX mice. Its superior efficacy over unfermented *S. fulvellum* suggests that fermentation significantly enhances the functional potential of this marine resource. Through its modulation of lipid profiles and normalization of adipocyte morphology, SfLlLm is a promising candidate for dietary intervention in postmenopausal women and others at risk of metabolic syndrome. While the OVX model provides translational relevance, caution is warranted in extrapolating the findings to humans due to species-specific differences in metabolism. In addition, while this study demonstrated the beneficial effects of SfLlLm on lipid metabolism and adipose tissue remodeling, the absence of molecular analyses limits mechanistic interpretation. Moreover, our study did not include mechanistic assays to identify the molecular targets or signaling pathways involved in the observed effects. Future studies should incorporate gene and protein expression analyses, such as those related to PPARγ (peroxisome proliferator-activated receptor gamma), adipogenic regulators, and lipid metabolism enzymes, to elucidate the underlying mechanisms of action. Future investigations should incorporate gene or protein expression analyses related to lipid metabolism, inflammation, and estrogen signaling to substantiate the proposed bioactive effects. Further clinical studies are needed to validate the efficacy and safety of fermented *S. fulvellum* as a nutraceutical for improving cardiovascular and metabolic health in postmenopausal populations.

## 4. Conclusions

This study demonstrates that fermented *S. fulvellum* (SfLlLm) significantly improves serum lipid profiles and mitigates adipose tissue hypertrophy in an estrogen-deficient OVX mouse model. Compared to its unfermented counterpart, SfLlLm exhibited superior efficacy in reducing triglycerides and LDL-C levels. Histological analysis further revealed that SfLlLm supplementation restored adipocyte size and distribution to levels comparable to those observed in the 17β-estradiol-treated group. These metabolic improvements are likely attributable to fermentation-derived bioactive compounds such as menaquinones, SCFAs, and microbial exopolysaccharides. Collectively, these findings support the potential application of fermented *S. fulvellum* as a functional dietary intervention for mitigating postmenopausal metabolic dysfunction. However, given the preclinical nature of the study, further molecular and clinical investigations are warranted to validate these effects in humans.

## Data Availability

The data presented in this study are available on request from the corresponding author. The data are not publicly available due to privacy or ethical restrictions.
